# A Rounded Approach to the Management and Treatment of Obstructive Lung Diseases

**DOI:** 10.3390/jcm11144240

**Published:** 2022-07-21

**Authors:** Silvano Dragonieri, Andras Bikov

**Affiliations:** 1 Respiratory Diseases, University of Bari, Piazza Giulio Cesare 11, 70121 Bari, Italy; 2 Wythenshawe Hospital, Manchester University NHS Foundation Trust, Manchester M13 9WL, UK; andras.bikov@gmail.com

Obstructive lung diseases are characterized by obstruction to airflow, inflamed and easily collapsible airways, and difficulties in exhaling, with a socio-economic burden in terms of medical visits and hospitalizations.

Even though the recent progress of scientific knowledge and the availability of new therapeutic and diagnostic tools have improved the management of obstructive diseases, they remain a foremost public health issue and a challenge for physicians.

Consequently, asthma and COPD are hot topics, as their high prevalence, morbidity, and mortality create many clinical challenges. In this Special Issue, we aimed to provide a rounded insight to the pathophysiology, diagnosis, and treatment of obstructive lung diseases.

A better understanding of pathophysiology of obstructive lung diseases is absolutely required. Interestingly, it is known that some forms of severe asthma and COPD may present similar characteristics and are difficult to distinguish. In this regard, Obojski et al. evaluated the intensity of the remodeling of the bronchi and the emphysema qualities of the lungs in patients with severe asthma with persistent airflow limitation (SA-PAL), COPD and healthy volunteers by quantitative computed tomography (QCT), showing that emphysema does not only occur in COPD, but also in SA–PAL [1].

Focusing on the development of emphysema, in their review, Jung et al. summarized the scope, risks, and limitations of current standard-of-care techniques including lung imaging and lung function and emerging diagnostics and monitoring tools (such as X-ray phase contrast, photoacoustic tomography, ultrasound computed tomography, electrical impedance tomography, forced oscillation technique, and impulse oscillometry system coupled with artificial intelligence and machine learning analysis), evidencing how regional lung function monitoring and the prediction of exacerbations and/or disease onset are fundamental to establish timely interventions to limit COPD progression to severe emphysema [2].

Acute exacerbations of COPD (AECOPD) are major events with potential serious consequences. Notably, AECOPD represents the leading cause of hospitalization and death in COPD patients. Neutrophil-to-lymphocyte ratio (NLR) is a hematological parameter that is an independent predictor of outcomes in stable COPD, although its prognostic role in patients with AECOPD is still matter of debate. Zinellu et al. [3] conducted a systematic review and meta-analysis of literature, indicating that in patients with AECOPD NLR on admission is significantly associated with the risk of severe adverse events during hospitalization, particularly short-term mortality up to 90 days [3].

It is now well known that COPD is associated with significant systemic abnormalities such as dysfunction of skeletal muscles, which affects motor function and limits the ability to carry out habitual activities of daily life. Casabona et al. used the spectral analysis of the electromyographic signal as a non-invasive approach to investigate the fiber muscle composition in COPD patients, reporting a good correlation between the changes in frequency–domain features and disease severity, thus suggesting that electromyography spectrum analysis applied to a non-fatigable motor task might be suitable for monitoring the progression of disease severity and the effects of physical rehabilitation programs [4]. In line with this, severe emphysema and respiratory muscle fatigue may lead to chronic hypercapnia, which is an independent risk factor of mortality in COPD. Csoma et al. focused on the pulmonary and systemic effects of hypercapnia and highlighted the effects of long-term non-invasive ventilation in COPD. Moreover, their review provides clinical recommendations and future research directions [5].

Taken together, these studies will help physicians in improving their understanding of obstructive lung diseases with up-to-date data, which might be of inspiration for forthcoming investigations.
